# Combining multiple features for error detection and its application in brain–computer interface

**DOI:** 10.1186/s12938-016-0134-9

**Published:** 2016-02-04

**Authors:** Jijun Tong, Qinguang Lin, Ran Xiao, Lei Ding

**Affiliations:** School of Information Science and Technology, Zhejiang Sci-Tech University, Hangzhou, 310018 China; School of Electrical and Computer Engineering, University of Oklahoma, Norman, OK 73019 USA; Center for Biomedical Engineering, University of Oklahoma, Norman, OK 73019 USA

**Keywords:** BCI, Error detection, Multi-channel, Combination of features

## Abstract

**Background:**

Brain–computer interface (BCI) is an assistive technology that conveys users’ intentions by decoding various brain activities and translating them into control commands, without the need of verbal instructions and/or physical interactions. However, errors existing in BCI systems affect their performance greatly, which in turn confines the development and application of BCI technology. It has been demonstrated viable to extract error potential from electroencephalography recordings.

**Methods:**

This study proposed a new approach of fusing multiple-channel features from temporal, spectral, and spatial domains through two times of dimensionality reduction based on neural network. 26 participants (13 males, mean age = 28.8 ± 5.4, range 20–37) took part in the study, who engaged in a P300 speller task spelling cued words from a 36-character matrix. In order to evaluate the generalization ability across subjects, the data from 16 participants were used for training and the rest for testing.

**Results:**

The total classification accuracy with combination of features is 76.7 %. The receiver operating characteristic (ROC) curve and area under ROC curve (AUC) further indicate the superior performance of the combination of features over any single features in error detection. The average AUC reaches 0.7818 with combined features, while 0.7270, 0.6376, 0.7330 with single temporal, spectral, and spatial features respectively.

**Conclusions:**

The proposed method combining multiple-channel features from temporal, spectral, and spatial domain has better classification performance than any individual feature alone. It has good generalization ability across subject and provides a way of improving error detection, which could serve as promising feedbacks to promote the performance of BCI systems.

## Background

The technology of brain–computer interface (BCI) has recently gained increasing attentions and has great potentials in improving the quality of life for people suffering from severe motor disabilities, such as cerebral palsy and paralysis [1]. The goal of BCI is to establish a communication channel between human brains and ambient environment [2], by directly decoding brain signals in order to control external devices. Electroencephalography (EEG) is one of the most popular measurement techniques used in BCI, which acquires electrical signals of the human brain with electrodes attached to the scalp. Comparing to other measurement techniques, EEG has the advantages of noninvasive, low cost, and easy setup. Therefore, it has been widely adopted in BCI technologies [3]. Brain patterns in EEG utilized in BCI applications include P300 [4, 5], motor imagery [6, 7], steady-state visually evoked potentials (SSVEP) [8, 9], error-related negativity (ERN) [10] and so on [11, 12].

While successful demonstrations have been achieved in laboratory settings, the application of BCI technologies in real-life scenarios still faces critical challenges. Due to its noninvasive nature, EEG recordings are relatively far from signal sources and are further smeared by the scalp, cerebrospinal fluid and other soft tissues sitting in between. These factors result in useful EEG signals that are usually weak and susceptible to static and electromagnetic interference, as well as other spontaneous activities, such as electromyography from movements of head and eyes [13]. These limitations of EEG make inevitable errors in the process of detecting users’ intentions in BCI systems [14]. It is thus of great importance in improving the robustness and reliabilities of BCI systems in order to achieve real-life applications.

Human and other species [15] learn and adapt their behaviors through the perception of errors. Past studies found that a time-locked negative deflection in EEG, mostly visible in frontal and central cortical sites, accompanies the occurrence of errors, namely, error-related negativity (ERN) [16]. Similar negativities in EEG signals have been reported in BCI studies when subjects observe incorrect outputs from BCI systems [17, 18]. The negative potentials detected at the onset of unexpected feedbacks (feedback ERN, or fERN) [19, 20] can be utilized to adjust command outputs of BCI systems. Thus, improvements on detection of error potentials (ErrPs) could facilitate the development of BCI systems with improved accuracy. Spuler et al. [21, 22] implemented an error–correction scheme in a P300 speller to correct error outputs in order to improve writing speed, which instantiates the application of ErrP detection in EEG data for promoting performance of BCI systems. However, both scanty knowledge about the neural mechanism of ErrPs and their temporal variations in status, amplitude and latency impose difficulties on the investigation [23].

The key factor in error detection is to effectively extract specific features from raw EEG data that are with abundant information, but of low signal-to-noise ratio. Various algorithms have been developed in searching for effective methods to extract characteristic features of ErrPs. Dal Seno et al. [24] proposed a genetic algorithm to extract features based on encoding different weight functions. Such algorithm is not only applicable to the extraction of P300 features, but also ERN signals. Omedes et al. [25] utilized low-frequency components as features on top of traditional feature extraction method in the temporal domain. Zhang et al. [26] came up with a method using directed transfer function (DFT) to extract continuous features that can improve the detection rate of error-related potentials, such as ERN. In term of spatial features, Ramoser et al. [27] proposed a spatial filtering method to extract features related to motor imagery in EEG, i.e., common spatial pattern (CSP). Such method searches for a set of weight coefficients at different channels of EEG to combine multiple-channel data to one, on which variance from different task conditions can be maximized in order to improve classification rate. Due to the vulnerability of CSP algorithm to overfitting, Song and Yoon proposed an adaptive CSP [28], Lotte and Guan investigated means in regularizing CSP [29], and Li et al. proposed L1-norm based CSP [30], all in an effort to overcome the overfitting problem. Shou and Ding [31, 32] proposed blind source analysis and studied EEG signals including ErrP associated with errors.

Because of the nonstationarity of EEG, no optimal features can be extracted from temporal or spectral domain alone. Meantime, due to the fact that various activities take place across different brain regions, overfitting might occur if using features from all channels for classification [33]. On the other hand, it is a critical challenge to select feature channels containing large inter-condition differences, without affecting the performance of BCI systems [34]. To tackle these problems, a procedure is proposed in the present study, which includes two times of dimensionality reduction on three types of features from temporal, spectral, and spatial domains with the use of neural network, and then the features are combined for classification. The present results from experimental data suggest superior classification performance of combined features over any individual features alone.

## Methods

### Experimental protocol

EEG data from the BCI challenge in IEEE EMBS NER 2015 conference were chosen for evaluation [35]. Perrins et al. designed the experimental protocol and collected the EEG data [36]. Twenty-six healthy subjects took part in this study (13 males and 13 females, mean age = 28.8 ± 5.4, range 20–37). All subjects went through five copy-spelling sessions. Each session consisted of 12 five-letter words, except the fifth which consisted of 20 five-letter words.

All subjects reported normal or corrected-to-normal vision and had no previous experience with the P300 speller paradigm or any other BCI applications. EEG data were recorded with 56 passive Ag/AgCl EEG sensors whose placement followed the extended international 10–20 system. Their signals were all referenced to a reference sensor at the nose. The ground electrode was placed on the shoulder and impedances were kept below 10 kΩ. Signals were sampled at 600 Hz.

In order to evaluate the generalization ability across subjects, the data from 16 participants was used for training and the rest 10 for testing.

### Preprocessing

The downloaded EEG data have been downsampled to 200 Hz.

Since previous literatures indicate that information of error related potentials mainly falls into the theta band and mu rhythm [17, 25], before further processing, we firstly used a fourth order Butterworth bandpass filter (1–20 Hz) to remove DC component and high-frequency noise [37]. After that, independent component analysis (ICA) was applied to filtered EEG data to remove common artifacts, such as eye movements, electrocardiography (ECG) and so on. EEG data from all channels were then referenced to a common average reference (CAR) to further increase signal-to-noise ratio [38]. At last, all data points of each epoch between 200 and 1000 ms after feedback onset were selected as one sample.

### Feature extraction

**Fig. 1:**
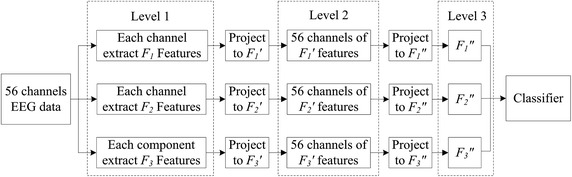
Feature extraction diagram

The features from temporal, spectral, and spatial domains were extracted from EEG signals, and then the back-propagation neural network (BP neural network) was adopted to perform two times of dimensionality reduction, in the end the acquired three levels of features from temporal, spectral, and spatial domains were used in another BP neural network for classification. The procedure is detailed in Fig. 1.

Step 1: Extract temporal feature *F*_*1*_ from each EEG channel as the level-1 features.
Step 2: Using the level-1 features *F*_*1*_ from the training group to train a BP neural network, which was applied to classify *F*_*1*_ features. The derived one-dimensional post-hoc probabilities were the level-2 features *F*_*1*_*′*.
Step 3: Using the level-2 features *F*_*1*_*′* from all channels to train another BP neural network, which was applied to classify the 56-dimensional level-2 features *F*_*1*_*′*, resulting in one-dimensional level-3 temporal features *F*_*1*_*′′*.
Step 4: Extract level 1 features *F*_*2*_ in the spectral domain and repeat step 2 and 3 to achieve one-dimensional spectral features *F*_*2*_*′′*.
Step 5: Extract level 1 features *F*_*3*_ from in the spatial domain and repeat step 2 and 3 to achieve one-dimensional spatial features *F*_*3*_*′′*.
Step 6: Combine three features [*F*_*1*_*′′ F*_*2*_*′′ F*_*3*_*′′*] from the training group to train a feedforward neural network, which was applied to classify samples from the testing group.

To extract the level-1 features at different domains, a series of algorithms were implemented and described as below.

①Extraction of the level-1 features in the temporal domain (*F*_*1*_): the training data were separated into two classes based on their labels, i.e., positive and negative feedbacks. $\bar{y} \in [\rm Position,Negative]$ denotes the mean of each class. Then the correlation *R*_*xy*_ and covariance *C*_*xy*_ between each sample *x* and $\bar{y}$ were computed as the feature set *F*_*1*_, using

1 $${R_{xy}}(m) = \left\{ \begin{array}{l} \sum\limits_{j = 0}^{N - m - 1} {{x_{j + m}}{{\overline y }_j}} ,\;\;\;m \ge 0\\ {R_{yx}}( - m),\;\;\;\;\;\;\;\;m < 0 \end{array} \right.$$

2 $${C_{xy}}(m) = \left\{ \begin{array}{l} \sum\limits_{j = 0}^{N - m - 1} {({x_{j + m}} - \frac{1}{N}\sum\limits_{i = 0}^{N - 1} {{x_i}} )({{\overline y }_j} - \frac{1}{N}\sum\limits_{i = 0}^{N - 1} {{{\overline y }_i}} )} ,\;\;m \ge 0\\ {C_{yx}}( - m),\;\;\;\;\;\;\;\;\;\;\;\;\;\;\;\;\;\;\;\;\;\;\;\;\;\;\;\;\;\;\;\;\;\;\;\;\;\;\;\;\;\;m < 0 \end{array} \right.$$

where *x* denotes each sample, *N* is the length of each sample and m is the corresponding latency.

②Extraction of the level-1 features in spectral domain (*F*_*2*_): the extraction was achieved following the approach from Huang et al. [39]. The empirical mode decomposition (EMD) was firstly performed to decompose samples from each channels into intrinsic mode functions (IMF) using

3 $$x(t) = \sum\limits_{i = 1}^{n} {c_{i} } + r_{n}$$

where *c*_*i*_ is IMF, *n* is the number of IMF decomposed, *r*_*n*_ is residue after EMD decomposition. Then Hilbert transformation was performed on each IMF component:

4 $$y_{i} (t) = \frac{1}{\pi }\int_{ - \infty }^{\infty } {\frac{{c_{i} (\uptau)}}{{t -\uptau}}} d(\uptau)$$

The analytic signal *z*_*i*_(t) was achieved by:

5 $$z_{i} (t) = c_{i} (t) + jy_{i} (t) = a_{i} (t)e^{{i\theta_{i} (t)}}$$

where *a*_*i*_(*t*) and *θ*_*i*_(*t*) were instantaneous amplitude and phase respectively, which were calculated by:

6 $$a_{i} (t) = \sqrt {y_{i}^{2} (t) + c_{i}^{2} (t)}$$

7 $$\theta_{i} (t) = \arctan \frac{{y_{i} (t)}}{{c_{i} (t)}}$$

Then instantaneous frequency of the ith IMF component was acquired by taking the derivative of *θ*_*i*_(*t*) as

8 $$\omega_{i} (t) = \frac{{d\theta_{i} (t)}}{dt}$$

Thus, signal *x*(t) can be describe as below in reflecting its changing amplitudes along time and frequency:

9 $$x(t) = \sum\limits_{i = 1}^{n} {a_{i} (t)e^{{j\int {\omega_{i} (t)dt} }} } = H(\omega ,t)$$

The Hilbert spectrum for each IMF component was denoted as:

10 $$H_{i} (\omega ,t) = a_{i} (t)e^{{j\int {\omega_{i} (t)dt} }}$$

Finally, relative energy coefficient (*E*), mean frequency ($\Phi$), mean slope (*MS*), and coefficient of variance (*CV*) were calculated as below to form the level-1 features in the spectral domain $F_{2} = \left[ {E_{i} \quad \varPhi_{i} \quad MS_{i} \quad CV_{i} } \right],\quad \left( {\text{i} \in 1,2, \ldots ,\text{n}} \right)$.

11 $$E_{i} = \frac{{\int_{ - \infty }^{\infty } {H_{i}^{2} (\omega ,t)d\omega } }}{{\sum\nolimits_{i = 1}^{3} {\int_{ - \infty }^{\infty } {H_{i}^{2} (\omega ,t)d\omega } } }}$$

12 $$\Phi _{i} = \frac{1}{N}\sum {\omega_{i} (t)}$$

13 $$MS = \frac{1}{N}\sum {\frac{{dc_{i} (t)}}{dt}}$$

14 $$CV_{i} = \frac{{\sigma_{i} }}{{\mu_{i} }}$$

where *μ*_*i*_ and *σ*_*i*_ are the mean and standard deviation of the ith IMF component.

③Extraction of the level-1 features in the spatial domain (*F*_*3*_): the extraction was implemented through four steps based on the approach from Ramoser et al. [27]

- Calculate the mean covariance matrices $\bar{R}_{p}$ and $\bar{R}_{n}$ for the two classes (positive and negative feedbacks), and eigenvalue decomposition as $\bar{R}_{p} + \bar{R}_{n} = U_{C} \lambda_{C} U_{C}^{'}$
- Calculate the whitening transfer matrix $P = \sqrt {\lambda_{C}^{ - 1} } U_{C}^{\prime }$
- Whitening transformation on the mean covariance matrix $S_{i} = P\bar{R}_{i} P^{T} ,i \in [n,p]$
- *S*_*n*_ and *S*_*p*_ share common eigenvectors B, i.e., *S*_*i*_ = *Bλ*_*i*_*B*^*T*^, *i* ∊ [*n*, *p*]
- Each row in the projection matrix *W* = *B*^*T*^*P* is the common spatial pattern of the two classes
- The feature set *F*_*3*_ consisted of *Y*_*i*_ = *W*_*i*_^*T*^*X*, (*i* ∊ 1, 2, …, 56).

While the common spatial pattern filter (each row of *W*_*56*×*56*_) provides a mathematical mean of combining features in the spatial domain, manual adjustment is still required to further improve the performance [33, 40]. Otherwise, overfitting could occur in classification due to the hyper-dimensional space [33]. However, in our method, we need not choose the filter manually, because we have realized the dimensionality reduction of spatial features using neural network from level 2 to 3 and could use all spatial filters, bypassing the redundant manual work.

### Dimensionality reduction

**Fig. 2:**
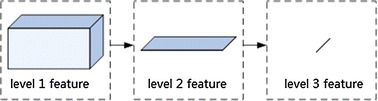
The procedure of dimensionality reduction

Each type of features was with different dimensionalities. There were 164, 12 and 161 dimensions in temporal, spectral and spatial features, respectively. Thus, the total length of the level-1 feature vector was 56 × (164 + 12 + 161). For convenience, we wrote it as 56 × 3 × *M*, where 56 was the channel number, the number 3 represented the number of kinds of features, and *M* ∊ [164, 12, 161] represented the length of corresponding features. The whole dimensionality reduction process is illustrated in Fig. 2. The 1st dimensionality reduction led to the collapse of level-1 features from a 3D space to level-2 features on a 2D plane, by replacing samples in level-1 features with posteriori probabilities. The dimension of the level-2 feature from all channels was then further collapsed, which could be visualized as the linearization of a plane (Fig. 2).

The feedforward BP neural network was used to reduce dimensionality of features. By inputting multi-dimensional level-1 features *F*, one-dimensional level-2 features *F′* were acquired after dimension deduction, by

15 $$F_{i}^{\prime } = {\text{tansig}}(W^{T} F_{i} + b)$$

where *i* ∊ [1, 2, 3] denotes different features. *W*^*T*^ and *b* are weights of the neural network and bias, respectively, acquired from training datasets. Tansig indicates the hyperbolic tangent sigmoid transfer function that calculated a layer’s output from its net input.

16 $${\text{tansig}}(n) = \frac{2}{{1 + e^{ - 2n} }} - 1$$

When repeating the same steps with level-2 features, the outputs were level-3 features as *F″*.

### Classification

For classification, the feedforward neural network was implemented after obtaining level-3 features *p* = [*F*_*1*_″*F*_*2*_″ *F*_*3*_″]. The neural network can be described as

17 $$Output = {\text{logsig}}(W^{T} p + b)$$

where *Output* is the classification results. *W*^*T*^ and *b* are weights of the neural network and bias, respectively, obtained from level-3 features *p* using training data. Logisg is a transfer function as

18 $${\text{logsig(n) = }}\frac{1}{{1 + e^{ - n} }}$$

## Results

### The features from different domains

*F*_*1*_, *F*_*2*_, *F*_*3*_ are features extracted from temporal, spectral, and spatial domains, respectively. The magnitude differences represent the ability to distinguish two types of signals.

**Fig. 3:**
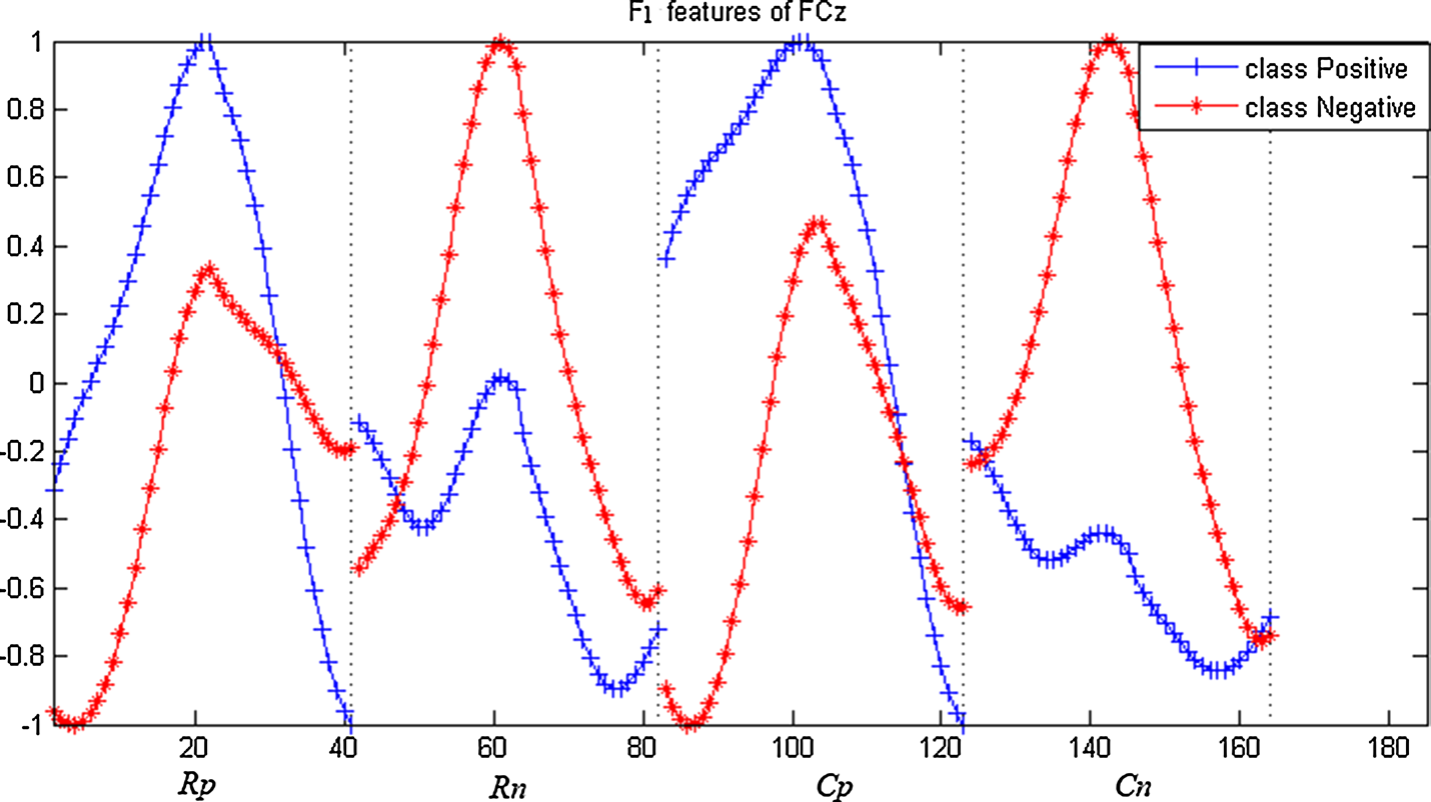
The grand average of temporal statistic characteristics F1 (Rp, Rn, Cp and Cn)

The feature set *F*_*1*_ consists of *R*_*p*_, *R*_*n*_, *C*_*p*_ and *C*_*n*_, and is shown in Fig. 3.

**Fig. 4:**
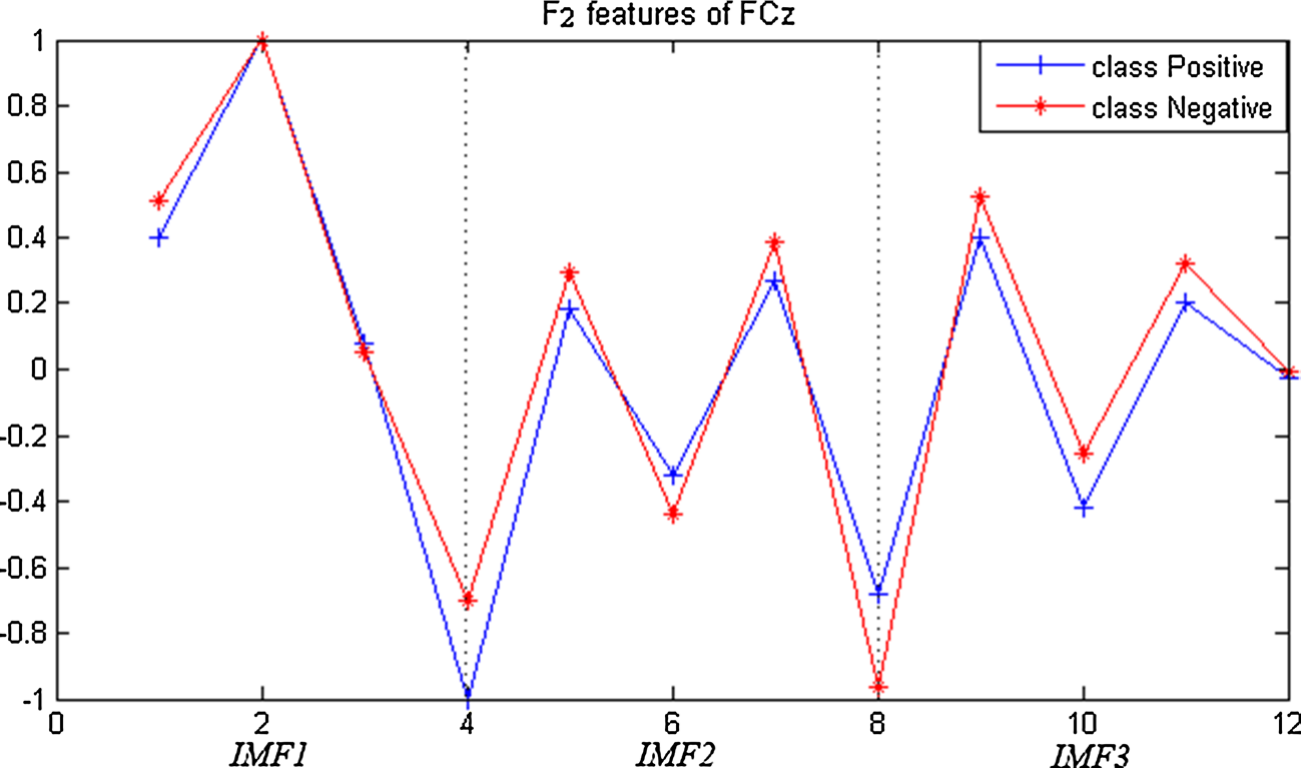
The grand average of F2 features (IMF1, IMF2, IMF3)

*F*_*2*_ is the intrinsic mode functions (IMF) decomposed by EMD decomposition. Samples from each channel are decomposed into four IMF components. Due to the reason that the fourth IMF is a monotonic curve, the first three components are chosen as *F*_*2*_ features, as shown in Fig. 4.

**Fig. 5:**
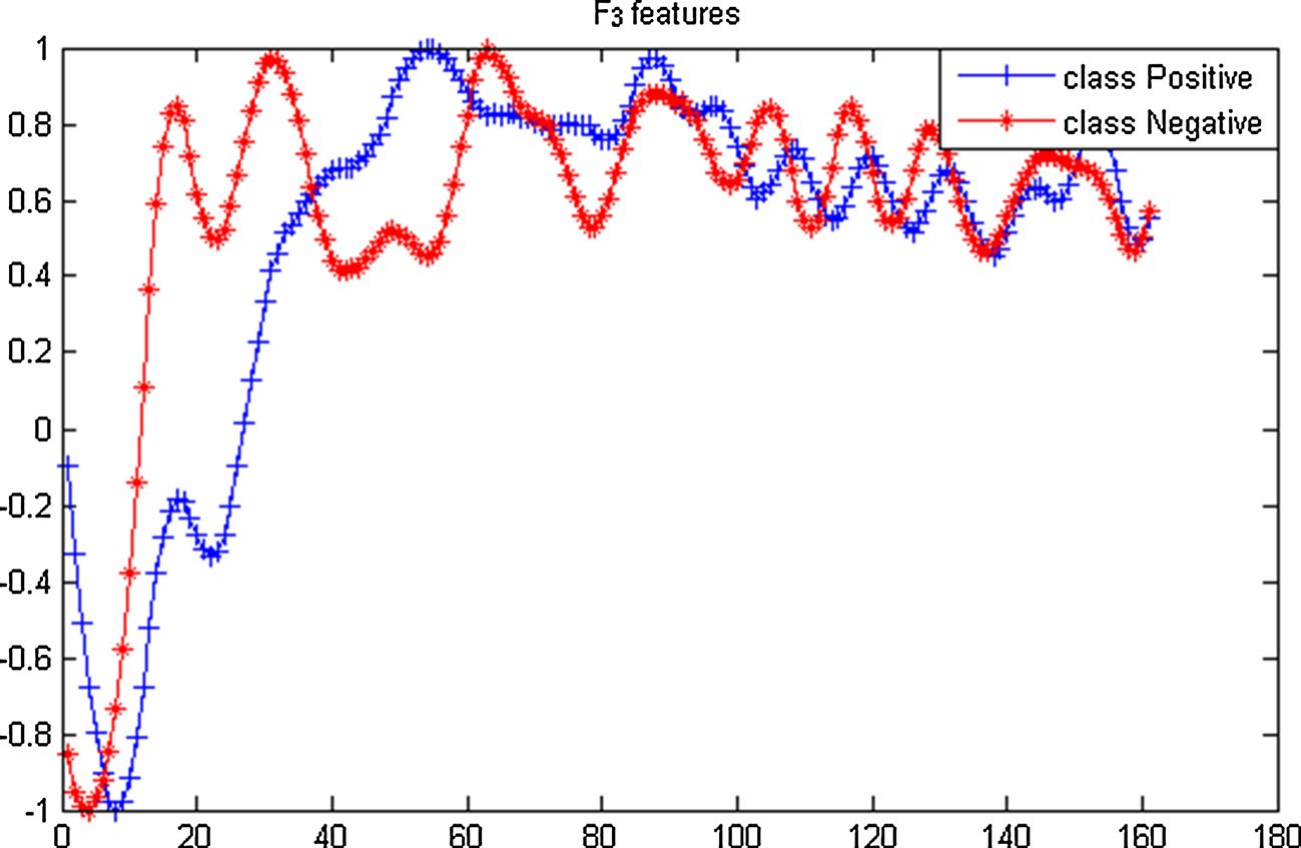
The grand average of projections of EEG onto the 1th eigenvector in B

*F*_*3*_ features are the projections of EEG from each channel on the projection matrix *W* from CSP. The projections of EEG onto the first or last eigenvector or some eigenvector in *B* were commonly used. Although that would decrease the difficulty, signal leakage could occur [41]. Figure 5 presents the projections of EEG onto the 1th eigenvector in *B.*

### Performance in error detection

The training and predict programs run on the personal computer (CPU: Intel(R) Core(TM) i5-4590 @ 3.30 GHz, RAM: 8 GB, System: Windows 10 64-bit, Platform: Matlab R2014a). The data from 16 participants (total 5400 samples) were used as the training set, which took 137 min for training. And it took 3.56 s to predict one sample in testing set.

**Fig. 6:**
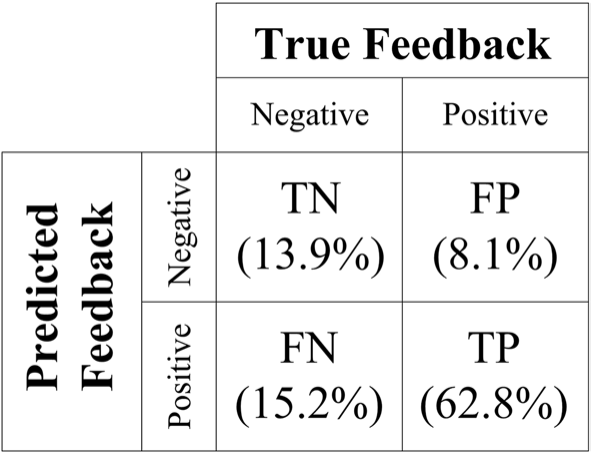
Confusion matrix for error detection. *TN* true negative; *FP* false positive; *FN* false negative; *TP* true positive (Testing group results)

The confusion matrix is implemented to evaluate the performance of classification. Figure 6 shows the results of multiple features (*F*_*1*_*′′* + *F*_*2*_*′′* + *F*_*3*_*′′*) from testing group including true negative (13.9 %), false positive (8.1 %), false negative (15.2 %) and true positive (62.8 %). The total accuracy is the sum of true positive and true negative, i.e., 76.7 %.

### Influence of features and individual variance

**Fig. 7:**
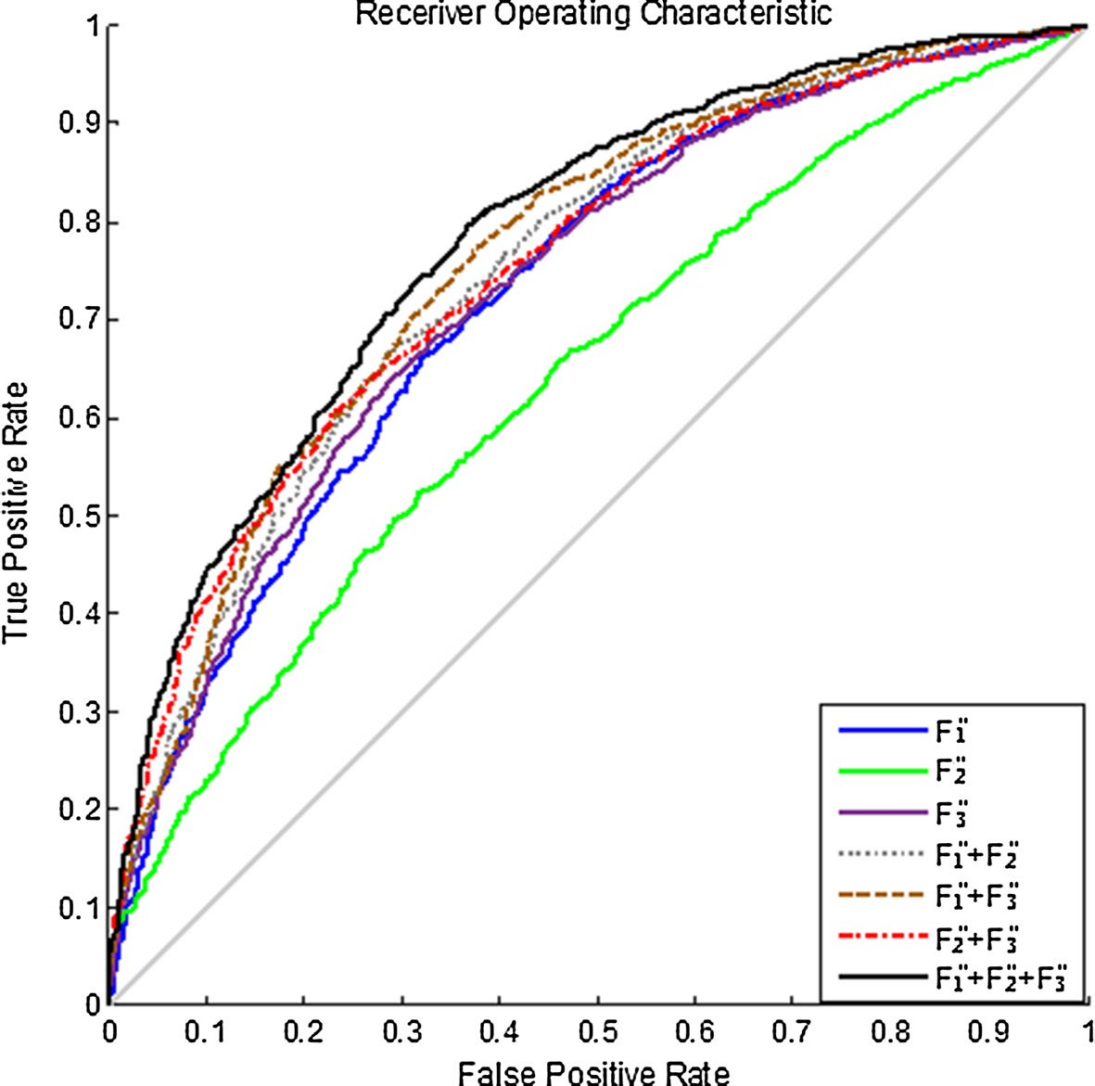
ROC curves from using different features (testing group result)

In order to further evaluate the effectiveness of feature extraction and the performance of classification, receiver operating characteristic (ROC) curves using different features and their combinations are shown as Fig. 7. *F*_*1*_, *F*_*2*_ and *F*_*3*_ represent the temporal, spectral, and spatial features, respectively. The combination of three features leads to the best performance. Table 1 shows the results of the variance among individual subjects for accuracies of error detection using the metric of area under ROC curves, from different types of features. It demonstrates the combination of features could improve the performance of classification, the results of a one-way Analysis of Variance (ANOVA) show significant difference (F = 7.24, p < 0.005) between single features and combination of three features.

**Table 1:** Individual AUC metric values from using different features

Subjects	*F*_*1*_ *′′*	*F*_*2*_ *′′*	*F*_*3*_ *′′*	*F*_*1*_ *′′* + *F*_*2*_ *′′*	*F*_*1*_ *′′* + *F*_*3*_ *′′’*	*F*_*2*_ *′′* + *F*_*3*_ *‴*	*F*_*1*_ *′′* + *F*_*2*_ *′′* + *F*_*3*_ *‴*
2	0.7475	0.6163	0.7493	0.7550	0.7908	0.7554	0.7990
3	0.8961	0.5662	0.8024	0.8638	0.8706	0.7992	0.8788
5	0.7497	0.6774	0.7870	0.7691	0.7896	0.8111	0.7968
6	0.7596	0.5642	0.7004	0.7486	0.7797	0.7071	0.7856
7	0.8169	0.6115	0.7843	0.8257	0.8381	0.7992	0.8531
8	0.7503	0.7161	0.9031	0.7878	0.8967	0.9238	0.8902
1	0.6547	0.5599	0.6280	0.6576	0.6507	0.6323	0.6645
4	0.6214	0.5950	0.5943	0.6382	0.6122	0.6146	0.6369
9	0.7430	0.5144	0.5488	0.7135	0.6184	0.5486	0.6617
10	0.6510	0.4981	0.6844	0.6246	0.6961	0.6570	0.6711
Average	0.7270	0.6376	0.7330	0.7501	0.7608	0.7520	0.7818

The data also reflect variance among individuals. Based on the classification performance from the combination of three features, the participants generally fall into two groups. One group contains 6 subjects with the average AUC of 0.8339, and another group contains 4 subjects with the average AUC of 0.6585. The performance using three single features in the first group also surpasses the second one, as shown in the first three columns of Table 1. One possible reason is that the participants in the first group were more concentrated in the task and the signal-to-noise ratios (SNR) of EEG data were higher than the second group during the extraction of useful features. Some previous studies have shown that electrophysiological responses are known to reflect participant’s involvements in the task [36, 42, 43].

### Influence of electrodes

Although multi-channel EEG signals could provide more comprehensive and complete information about different conditions, the added dimensionalities would also lead to overfitting and reduced the classification performance.

**Fig. 8:**
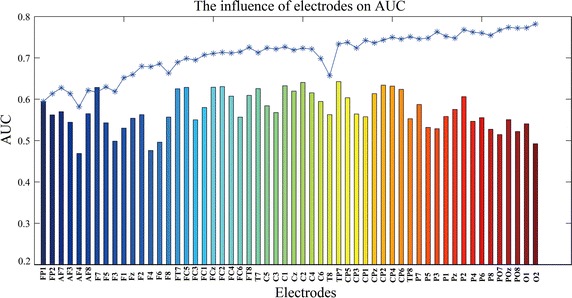
The influence of number of electrodes on AUC. Each bar denotes AUC of features from only one electrode. Each star presents AUC of combined features from FP1 to each of the following electrodes

Figure 8 presents the influence of electrodes on the classification performance from using the combination of three features. Firstly, it reveals the effect of electrode location to the classification results. Features from electrodes in central brain regions generally exceed those at peripherals in classification performance. Secondly, it shows that single electrodes have poor performance in detecting errors with the average AUC of 0.5726. The AUC values of features from electrodes (AF4, F4, F6, O2) are near 0.5, which demonstrate the poor ability of classification. With added features from more electrodes, the classification generally show increasing pattern except for a few electrodes (i.e., AF4, F8 and T8), illustrated by stars in Fig. 8. The added features lead to the adjustment of weights in neural network toward desired directions, which in turn contribute to the improvements in classification performance.

## Discussion

Feature extraction and representation are critical factors in error detection. Single features from temporal, spectral, and spatial domain have been widely investigated in many studies [25, 27]. In the present study, we proposed a method of error detection using neural network to combine various features from multiple-electrode EEG, which not only combines features of different domains, but also addresses the overfitting issue caused by the curse of dimensionality. In the contest, our performance score was 0.7818 and ranked fourth among all the 260 teams attending the challenge, as shown in Table 2.The abilities in error detection of the three features *F*_*1*_, *F*_*2*_, *F*_*3*_ can be observed in Figs. 3, 4, and 5, respectively, revealed by the magnitude differences in various features. The observation is in line with the classification performance in Fig. 7 and Table 1. For example, the magnitude differences are small for *F*_*2,*_ comparing to other two features, and its classification performance are also worse than others as shown in the 3rd column in Table 1. Nevertheless, there is still constructive information in *F*_*2*_ for error detection, suggested by the improved detection performance with *F*_*2*_ added to the combination of features in Table 1. Such combinations make use of information from temporal, spectral, and spatial domains, and provide more comprehensive information about errors than individual features. However, simple combination of features would result in long feature vectors. When further considering added information from different electrodes, the complex model is very susceptible to the overfitting problem in classification, which might lead to degeneration in performance. Therefore, feature extraction and dimensionality reduction play important roles in error detection in the present study. The feedforward BP neural network is implemented to reduce the dimensionality of features. The outputs of neural network are essentially the posterior probability of the primary inputs, and the values are between [0, 1] (values close to 1 favor the labeling towards positive class, and 0 to the negative class). After two times of dimensionality reduction, the level-3 features become just one dimension.

**Table 2:** The score and ranking of our method

Rank	AUC
1	0.8722
2	0.8566
3	0.8180
Our method	0.7818
5	0.7692
6	0.7479

In previous studies, some researchers realized dimensionality reduction through channel selection. They selected electrodes via observing topographic EEG power maps over the scalp [36, 37]. In addition, there are some other studies that implement PCA [44], ICA [45, 46] or other channel selection algorithms [39], for the selection of spatial features.

In term of detection performance, the following factors pose impacts in the proposed method. The first factor is the preprocessing of raw EEG data, such as removal of eye artifacts, time window length and cutoff frequency of bandpass filter. It is found that eye-movement artifact removal during EEG preprocessing could enhance the accuracy about 2 %. Another factor is the feature extraction process, such as the selection of time delay parameter *m* in the process of temporal feature extraction. The larger the m value is, the more information about error detection in *F*_*1*_ features. When extracting features in the spatial domain, it is found that other spatial filter method such as xDAWN [47] could also be used to improve performance.

The error detection is essentially a binary classification problem. Such type of classification usually suffers greatly from unbalanced sample numbers from different classes. This imbalanced sample numbers result in biased classification towards the majority class and lower detection rate in the minority class [48, 49]. To tackle such a problem, different techniques were explored to compensate inter-class sample differences, such as over-sampling and under-sampling [50]. In addition, some researchers improved the prediction rate of the minority class by adopting classifier algorithms [51]. In future works, it could be an important aspect to investigate in order to improve the accuracy of error detection.

## Conclusions

In the present study, to capture the discriminative information about error potentials in features from different domains and avoid overfitting caused by features of multiple dimensionalities, we proposed a new approach of combining multiple-channel features from temporal, spectral, and spatial domains through two times of dimensionality reduction based on neural network. It took advantage of information from multiple electrodes and combination of features from different domains rather than single features. The classification results using ROC curves and AUC metrics suggest superior performance with combined features over single features, and show the good generalization ability across subjects of the proposed algorithm. The improved accuracy in error detection in present study demonstrate great potentials in promoting the performance for BCI systems integrated with scheme of error correction. This could facilitate developing robust BCI systems towards real-life environment.
